# Giant mucinous cystadenocarcinoma of ovary in a young woman: a case report and review of literature

**DOI:** 10.3389/fonc.2024.1377042

**Published:** 2024-05-28

**Authors:** SiHong Zhu, MeiZhen Yao, LingNa Xiong, BuZhen Tan

**Affiliations:** Department of Obstetrics and Gynaecology, The Second Affiliated Hospital of Nanchang University, Nanchang, China

**Keywords:** ovarian epithelial tumor, mucinous cystadenocarcinoma, gynecology, young woman, case report

## Abstract

In April 2023, we successfully treated a 21-year-old patient afflicted with a rare giant cystadenocarcinoma, an extraordinarily large mucinous ovarian tumor that weighed nearly 25 kg. The preoperative dimensions of the tumor measured 40 × 30 × 34 cm, with the tumor’s weight nearing 25 kg. Despite its uncommon nature, we elected to perform a right adnexectomy, greater omentectomy, and peritoneal biopsy during the surgical intervention due to the patient’s youth and the family’s expressed desire to preserve fertility. In the subsequent August follow-up, CT scans revealed the complete resolution of the tumor, accompanied by the normalization of tumor markers, indicating a favorable outcome.

## 1 Introduction

Mucinous ovarian cancer (MOC) is one of the histological subtypes of epithelial ovarian carcinoma with unique clinical, histological, and molecular characteristics,the incidence of this type of ovarian cancer ranges from 3% to 5% (1, 2). MOC is common in women aged between 20 and 50 years, and the average age of onset is lower than that for serous cystadenocarcinoma; 80% of the patients are diagnosed with stage I ranges from 54% to 80% and usually present with a large solid cystic mass in the pelvic cavity, with a median diameter of 18 cm (5–48 cm). Patients with large masses may have symptoms of compression, such as frequent/urgent urination, urinary retention, dysuria, difficulty defecating, and constipation, and in severe cases, patients may experience urinary tract or intestinal obstruction. The late symptoms are lower abdominal discomfort, abdominal success, ascites, poor appetite, etc.

## 2 Medical history

The patient was a 21-year-old female college student with height and weight on admission of 183 cm and 100 kg, respectively. She was admitted to our hospital on April 22, 2023, with a complaint of “abdominal distension for 2 months that had aggravated since 1 week.” The patient self-reported menarche at the age of 14 with an irregular menstruation cycle of 5–6/20–40 days, no menstruation on April 20, 2023, and no dysmenorrhea. She denied any sexual history. Since February 2023, the patient experienced abdominal distension, nausea, poor appetite, constipation, and discomfort; frequency of urination was normal. The patient’s appetite significantly decreased as her abdomen expanded, leading to a reduced food intake; however, she did not exhibit any attempts to intentionally lose weight. In 2 months, she experienced weight loss of >10 kg.

## 3 Preoperative examination

Physical examination on admission revealed stable vital signs, whole abdomen distension, muscle tension, and no obvious tenderness rebound pain. Speculum examination of the anus and vulva revealed no abnormality. A huge mass was observed in the pelvic cavity; its upper margin of the mass was in contact with the xiphoid process; the mass had a clear boundary and was encased in a solid sac; no tenderness was observed.

Routine blood exam on admission revealed the following: platelet count, 515 × 109/L; fibrinogen concentration, 6.01 g/L; prothrombin time, 15.7 s; international standard ratio, 1.40; prothrombin activity, 64.3%; D-dimer level, 2.99 mg/L; serum carcinoembryonic antigen, 17.42 ng/mL; carbohydrate antigen-199, 28806.09U/mL; carbohydrate antigen-125, 138.70 U/mL; human epididymal protein 4, 227 pmol/L.

**Figure 1:**
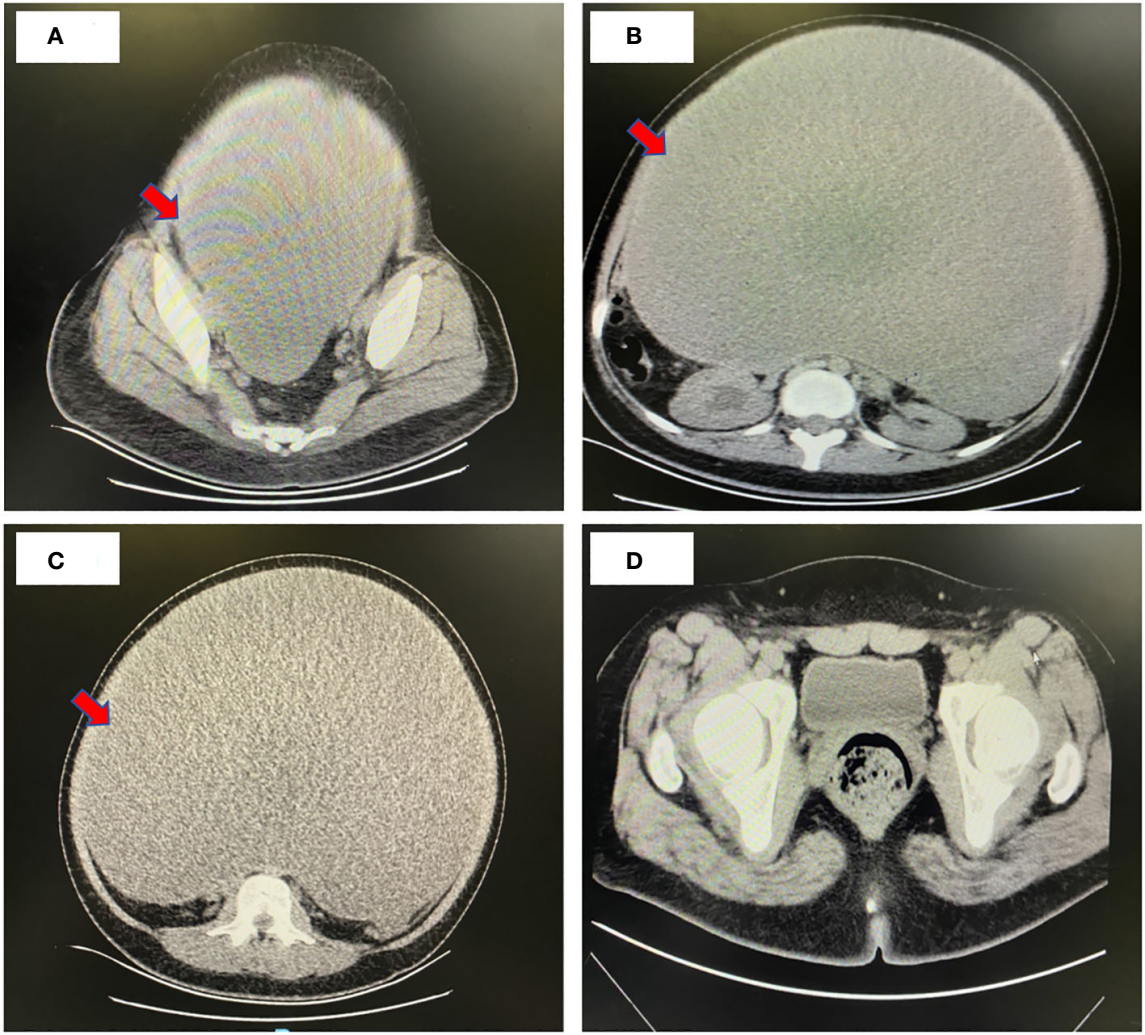
Whole-abdomen enhanced computed tomography image. **(A-C)** Preoperative CT findings: Abdominal distension and a substantial cystic fluid lesion in the abdomen measuring approximately 40 cm x 30 cm x 34 cm. Contrast enhancement revealed mild strengthening of both septum and capsule wall, with no internal fluid density shadow enhancement at any stage. No distinct limited thickening of the septum was observed. Uterine compression and leftward displacement, along with compression of the abdominal aorta and adjacent intestinal lumen, were noted. Right renal corpuscle and calyces exhibited dilatation and hydronephrosis, as did the upper part of the right ureter. Provisional diagnosis: Massive cystic presence in the abdominopelvic cavity, suggestive of a cystic tumor likely of right adnexal origin, possibly a plasma cystadenoma. Right upper ureter compression was implicated in right lower ureter dilatation and hydronephrosis. **(D)** Postoperative CT findings: Changes in the right ovary, with a few striated shadows and effusion in the operative area. Discernible uterine morphology, and the left adnexa appeared replete.

Whole-abdomen computed tomography (CT) revealed abdominal distention, the presence of a large cystic fluid-filled mass in the abdomen, measuring about 40 × 30 × 34 cm, separated cystic cavity visible inside; slight enhancement of the division and cyst wall was observed, but no enhancement was observed in the internal fluid shadow at each stage, and no significant localized thickening was observed in the division (**Figure 1**). The lower posterior wall was about 2 cm in diameter, with nodular and heterogenous, isodense masses on the right posterior wall. No enhancement was observed at any stage. Uterine compression with left displacement, and no obvious abnormal shadow and compression of the abdominal aorta and adjacent intestinal cavity were observed. Dilatation and hydrops of the right renal calyces and the right upper ureter were observed. No abnormal shadow was found in the left renal parenchyma, and no water was found in the left kidney. No effusion was observed in the abdomen and pelvis, and no significantly enlarged lymph nodes were observed retroperitoneally. Right upper ureter occlusion, right hydronephrosis, and pressure on the right lower ureter were observed. The diagnosis of large cystic mass in the abdomen and pelvic cavity was established. The possibility of a cystic tumor originating in the right adnexa and serous cystadenoma was considered.

**Figure 2:**
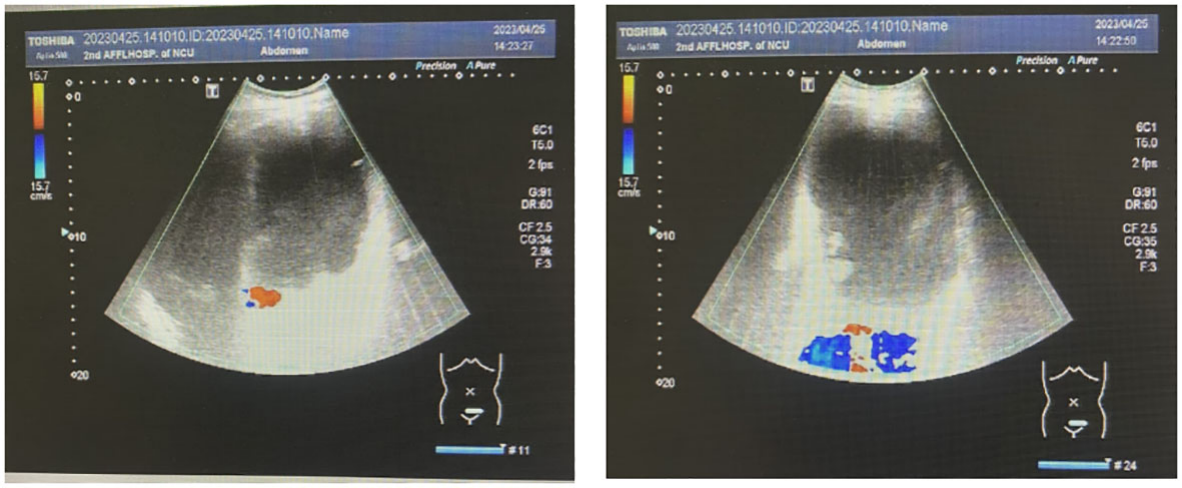
Abdominal color ultrasonography scans.

Abdominal color ultrasonography showed the presence of a large cystic mass in the abdominal pelvis and a polyp in the gallbladder. The spleen was enlarged (**Figure 2**). Color ultrasonography of the urinary system showed mild hydronephrosis of the right kidney and widening of the upper segment of the right ureter, with obstruction of the middle and lower segments, not due to the compression by the large huge mass. No obvious abnormalities were found in the left kidney, left ureter, and bladder.

Gynecologic color ultrasonography suggested the presence of a serous cystadenoma originating from the right ovary. Electronic gastroscopy showed chronic gastritis esophagitis, and electronic colonoscopy showed no abnormality.

## 4 Intraoperative exploration

**Figure 3:**
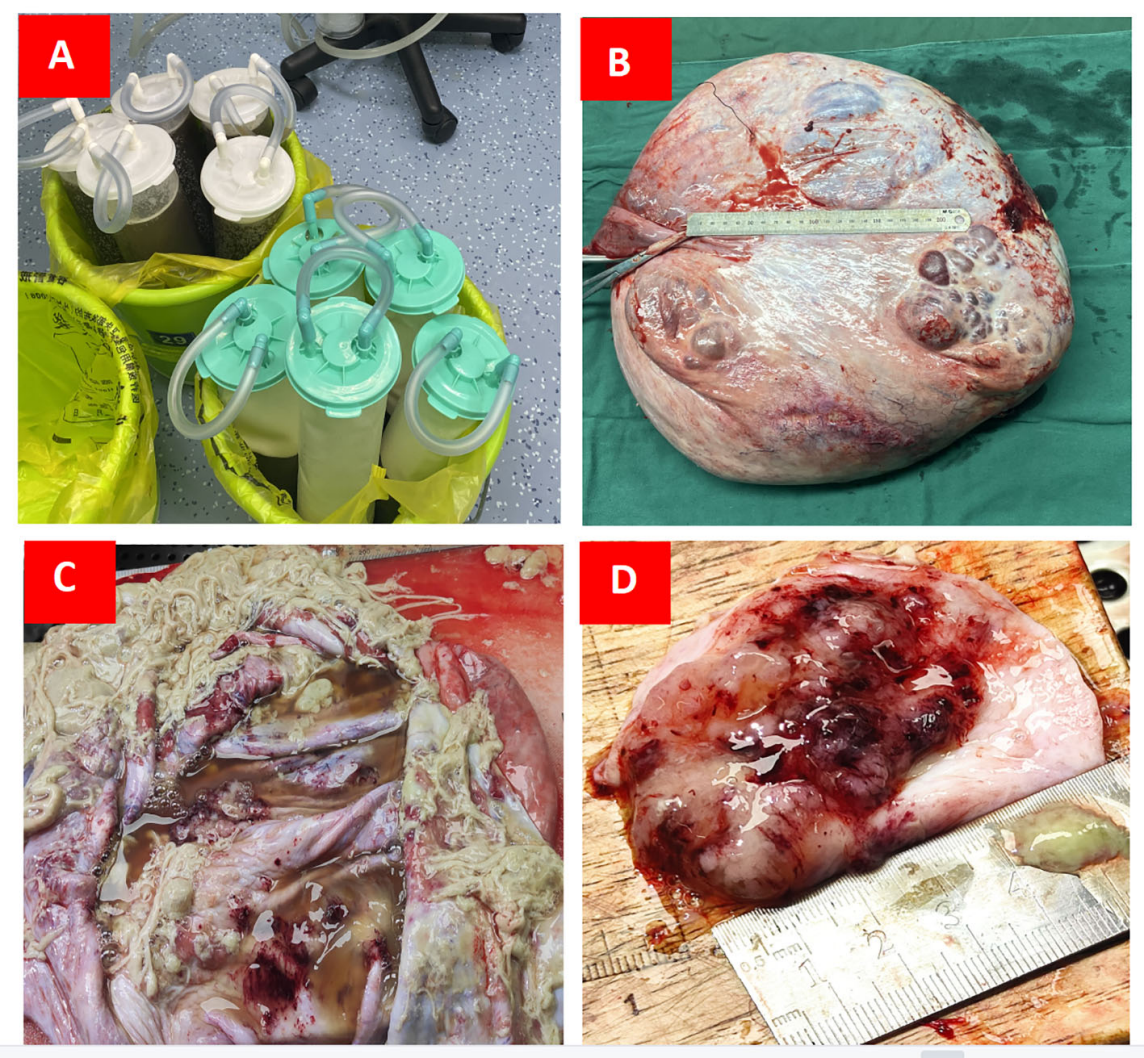
**(A)** Approximately 14,000 mL of sac fluid was aspirated intraoperatively; **(B)** The cyst size measured about 40 × 35 × 15 cm after draining the fluid from the sac; **(C)** The internal structure of the cyst was examined on a section prepared postoperatively; **(D)** Internal solid area of cyst was 5 × 5 × 1.5 cm.

After the preoperative examinations, an exploratory laparotomy was performed on April 26, 2023. Intraoperative exploration showed the presence of a large cystic mass in the pelvic cavity; the upper boundary of the mass was close to the xiphoid process, and it was tightly adhered to the anterior peritoneum, lateral peritoneum, and greater omentum. Because the tumor completely occupied the dorsal cavity of the abdomen, its source could not be identified. First, about 14,000 mL of liquid was aspirated from the clear capsule. Then, on exploring the pelvic cavity, it was found that the mass originated from the right ovary; the right fallopian tube was attached to the mass, the uterus was anteriorly positioned and enlarged, and the appearance of the left adnexa was normal. During the operation, 300 mL of the flushed ascites solution was reserved for examination. Peritoneal hyperemia was detected; proliferative nodules and follicular cysts were observed in the omentum; no obvious abnormalities were observed in the appearance of the liver, stomach, diaphragmatic surface, and appendix; the spleen was slightly enlarged; and pelvic lymph node enlargement was not detected. The right adnexa, measuring about 40 × 35 × 15 cm (after aspiration of 14,000 mL of intracapsular fluid), was completely removed. Tumor growth was observed on the surface. Left and right peritoneal tissues and lesion-like tissues of the omentum were retained for examination (**Figure 3**). The family was informed of the intraoperative situation, and the right appendage was removed first. Intraoperative frozen sections showed the presence of a borderline myxoid tumor (right ovary). Bilateral peritoneal tissue showed hyperplasia of local mesothelial cells. No tumor cells were found in the frozen section of the retinal tissue. Intraoperative diagnosis of “borderline mucinous tumor of the right ovary” was made. Due to the patient’s young age, her family expressed a desire to preserve her fertility following discussions with the patient. Consequently, during the operation, a recommendation was made for right adnexectomy, greater omentectomy, and peritoneal biopsy. In the follow-up examination in August, CT scans revealed resolution of the mass, and tumor markers returned to normal.

## 5 Postoperative pathology

**Figure 4:**
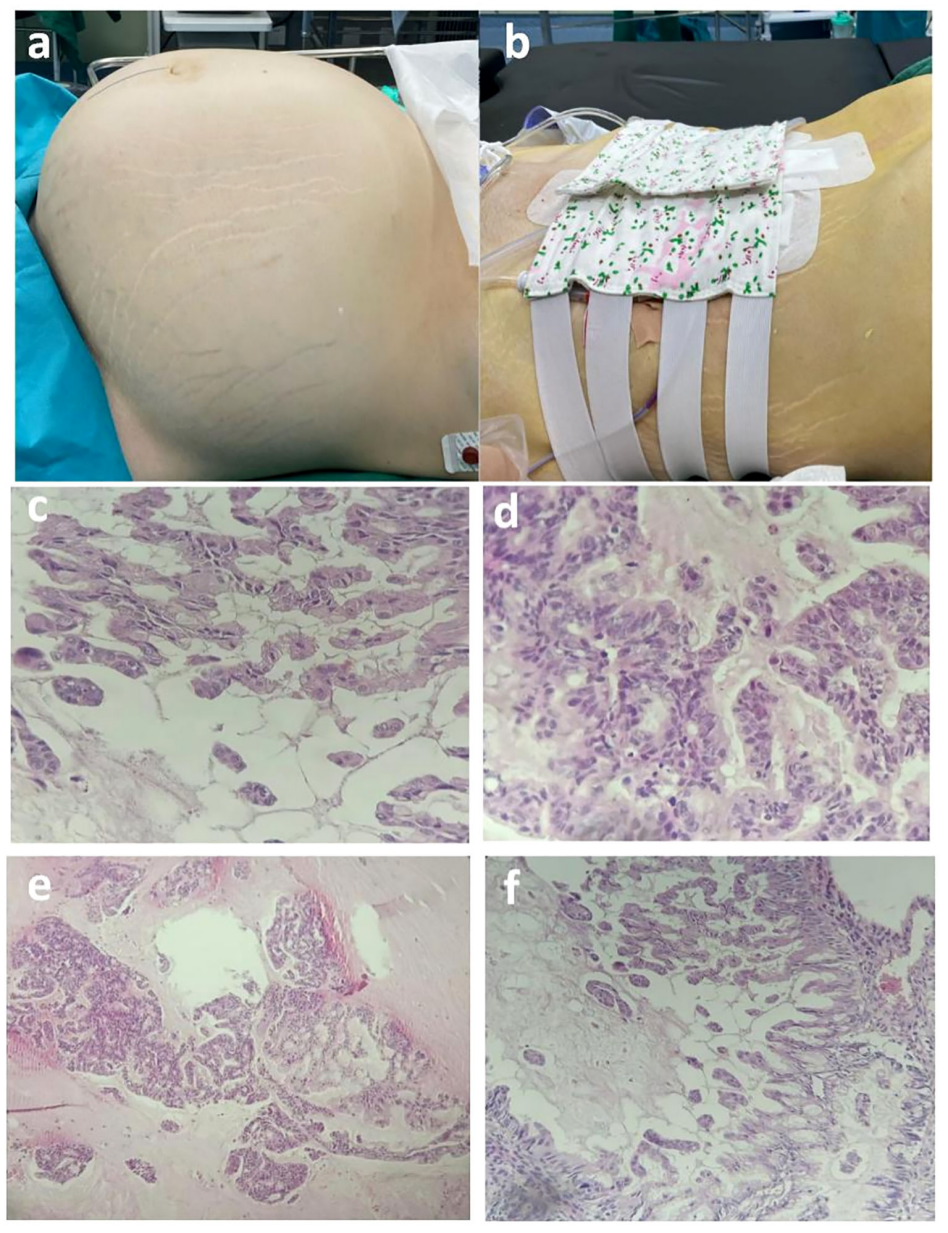
**(A, B)** Preoperative and postoperative comparison (preoperative abdominal circumference, 125 cm; weight, 98 kg; postoperative abdominal circumference, 90 cm; weight 74 kg)**. (C-F)** Postoperative pathological images of paraffin wax. Postoperative paraffin pathology revealed that the cyst wall exhibited a single layer of columnar epithelium, with intracellular mucus observed. In certain areas, sieve-like, labyrinthine, and papillary epithelial hyperplasia was evident, accompanied by increased cell layers, disorganized polarity, and noticeable anisotropy. Nuclear schizophrenic images were also observed. The pathological diagnosis based on the paraffin section report identified mucinous cystadenocarcinoma of the expansion infiltration type in the right ovary.

Histological examination of the mass specimen obtained during surgery showed that the capsule wall was lined with a single columnar epithelium and showed intracellular mucous with epithelial hyperplasia in some areas; cribriform, labyrinth like and papillary structures, were observed, with increased cell layers, disordered polarity, obvious atypia, and visible mitotic figures (**Figure 4**). Pathological examination of the paraffin section of the right ovary showed mucinous cystadenocarcinoma of the swelling and infiltrating type. No cancer tissue involvement was observed in the right fallopian tube, bilateral peritoneal tissue, and greater omentum. Pathological examination of the ascites showed no malignant tumor cells. The ascites cells showed no abnormal mitotic changes. The postoperative diagnosis was right ovarian mucinous cystadenocarcinoma of IC_2_ stage(T1c1N0M0) (3).

## 6 Discussion

Epithelial ovarian tumors account for 50–70% of primary ovarian tumors and 85–90% of ovarian malignancies, which are more common in middle-aged and elderly women than in pre-puberty and infants (4). Most mucinous cystadenocarcinomas are metastatic cancers, while primary mucinous cystadenocarcinomas are very rare, accounting for about 3–4% of all ovarian cancers (5).

Mucinous cystadenocarcinomas are usually unilateral ovarian masses with a large volume, smooth surface, multi-locular cysts/solid regions, and cystic components; they may be accompanied by bleeding and necrosis. Microscopically, the heterogeneous mucinous epithelium is arranged in a glandular or papillary formation, accompanied by fusion or destructive infiltration in the surrounding tissue. The pathology can be divided into two types: dilatation type and infiltration type. At present, it is believed that the occurrence of mucinous cystadenocarcinoma is a continuous change process from benign to borderline to malignant, showing a “stepped-progression pattern.”

Giant ovarian tumor refers to a tumor with a diameter greater than 20 cm. Due to its huge size, it is often associated with obvious symptoms of compression of neighboring organ systems. Clinically, the digestive, urinary, and respiratory systems are the most commonly compressed systems. In this case, the patient also showed significant abdominal distension, chest tightness, nausea, poor appetite, constipation, and discomfort. Color-Doppler ultrasonography and CT of the urinary system also showed mild hydrops of the right kidney, widening of the upper segment of the right ureter, obstruction of the middle and lower segments of the urinary system, and other signs of urinary compression.

Gynecologic ultrasonography is the first choice for the initial evaluation of ovarian tumors, and tumor markers such as serum CA-125, carcinoembryonic antigen (CEA), CA-125, CA19–9, and human epididymal protein (HE4) are also of significance in confirming the diagnosis of ovarian tumors. If tumor marker levels are abnormally elevated, the possibility of malignant tumor should be highly suspected (3, 4, 6, 7). CT is also important for preoperative evaluation, as it can determine the origin of the tumor, size, boundary, internal structure, and anatomic relationship with the surrounding tissues. In this case, the patient had the following marker levels: CA-199, 28806.09 U/mL; CEA, 17.42 ng/mL; CA-125, 138.70 U/mL; and HE4, 227 pmol/L. Whole-abdomen enhanced CT showed that the tumor volume was about 40 × 30 × 34 cm, and fine line separation was observed in the tumor. Slight enhancement of the partition and the capsule wall was observed, internal fluid showed no enhancement at any stage, and no significant localized thickening was observed in the partition. The lower posterior wall was about 2 cm in diameter and nodular, with heterogenous, isodense masses on the right posterior wall. No enhancement was observed at any stage. In general, when a tumor is removed surgically, the levels of its tumor markers also decrease. In our patient, the levels of tumor markers returned to normal 2 weeks after surgery.

Owing to the pressure of the tumor on important blood vessels such as abdominal aorta and inferior vena cava, hemodynamic abnormalities may occur (8). Upon admission, the patient was in a hypercoagulable state, with a platelet count of 515 × 109/L, fibrinogen concentration of 6.01 g/L, prothrombin time of 15.7 s, international standard ratio of 1.40, prothrombin activity of 64.3%, and D-dimer level of 2.99 mg/L. Her platelet and coagulation indexes returned to normal 2 weeks after surgery and anticoagulant therapy. According to the latest guidelines for the diagnosis and treatment of ovarian cancer, fertility preservation can be considered in patients with stage I ovarian cancer. In cases where intraoperative freezing confirms mucinous cancer and there are no suspicious or enlarged lymph nodes, it is recommended not to remove the lymph nodes. Regarding postoperative management, patients with cancer of stages IA–IB can be observed, those with stage IC can be observed or administered chemotherapy, and those with stages I–IV require systemic treatment (9, 10). The presence of borderline mucous tumor was initially considered due to the results of intraoperative frozen section of biopsy of the right ovary. The patient was young and desired to preserve her fertility function. Right adnexectomy, omentectomy and peritoneal biopsy were performed during the operation. Examination of postoperative paraffin section of the mass showed that it was mucinous cystadenocarcinoma of the right ovary of stage IC_2_(T1c1N0M0); therefore, oxaliplatin intravenous chemotherapy plus oral capecitabine therapy was started after surgery.

Due to early detection, diagnosis, and treatment, giant ovarian tumors are becoming increasingly rare. In 2023, Tomasz Kluz et al. (11) documented a case involving a large borderline mucinous cystadenocarcinoma of the ovary. The tumor presented as a multiloculated cyst, measuring 80 × 50 × 50 cm. Shrestha et al. (12) reported a case of giant MOC in 2022, measuring about 29 × 16 × 10 cm. The patient in our case was only 21 years old, and the tumor volume before surgery was about 40 × 30 × 34 cm, and the tumor weight was nearly 25 kg, significantly exceeding the tumor volume previously reported. Intraoperatively, it was found that the tumor originated from the right ovary, and no primary MOC lesions were found in other parts of the body. To our knowledge, this is the first report of giant primary mucinous ovarian cancer of such a large volume. Considering this case, we recommend adolescent women routinely undergo gynecological color ultrasonography every year to ensure early detection, diagnosis, surgery, and management of ovarian tumors.

## Data availability statement

The original contributions presented in the study are included in the article/supplementary material. Further inquiries can be directed to the corresponding author.

## Ethics statement

The studies involving human participants were reviewed and approved by Ethics Committee of the Second Affiliated Hospital of Nanchang University. The patients/participants provided their written informed consent to participate in this study and to the publication of this case report.

## Author contributions

SZ: Writing – original draft, Writing – review & editing. MY: Conceptualization, Data curation, Formal analysis, Funding acquisition, Investigation, Methodology, Project administration, Resources, Software, Supervision, Validation, Visualization, Writing – original draft. LX: Conceptualization, Data curation, Formal analysis, Funding acquisition, Investigation, Methodology, Project administration, Resources, Software, Supervision, Validation, Visualization, Writing – original draft. BT: Writing – original draft, Writing – review & editing.
